# The impact of tumor microenvironment: unraveling the role of physical cues in breast cancer progression

**DOI:** 10.1007/s10555-024-10166-x

**Published:** 2024-01-19

**Authors:** Ayuba Akinpelu, Tosin Akinsipe, L. Adriana Avila, Robert D. Arnold, Panagiotis Mistriotis

**Affiliations:** 1 Department of Chemical Engineering, Samuel Ginn College of Engineering, Auburn University, Auburn, AL 36849 USA; 2 Department of Biological Sciences, College of Science and Mathematics, Auburn University, Auburn, AL 36849 USA; 3 Department of Drug Discovery and Development, Harrison College of Pharmacy, Auburn University, Auburn, AL 36849 USA

**Keywords:** Breast cancer mechanobiology, Physical signals, Tumor microenvironment, Metastasis

## Abstract

Metastasis accounts for the vast majority of breast cancer-related fatalities. Although the contribution of genetic and epigenetic modifications to breast cancer progression has been widely acknowledged, emerging evidence underscores the pivotal role of physical stimuli in driving breast cancer metastasis. In this review, we summarize the changes in the mechanics of the breast cancer microenvironment and describe the various forces that impact migrating and circulating tumor cells throughout the metastatic process. We also discuss the mechanosensing and mechanotransducing molecules responsible for promoting the malignant phenotype in breast cancer cells. Gaining a comprehensive understanding of the mechanobiology of breast cancer carries substantial potential to propel progress in prognosis, diagnosis, and patient treatment.

## Introduction

According to the Centers for Disease Control and Prevention, breast cancer is the second leading cause of death among women in the US. Breast cancers are often classified based on their invasive potential. Ductal carcinoma in situ is a non-invasive type of breast cancer characterized by the uncontrolled proliferation of neoplastic epithelial cells, which remain localized within the mammary ducts [1]. In contrast, invasive breast cancers originate primarily in the milk ducts (invasive ductal carcinoma) or breast lobules (invasive lobular carcinoma) and can spread to nearby breast tissues or even metastasize to other body parts [1, 2]. Breast cancers can also be categorized into different subtypes based on the expression levels of the human epidermal growth factor receptor 2 (HER2) and two hormone receptors: estrogen receptor (ER) and progesterone receptor (PR). Luminal-like tumors (luminal A and luminal B) make up ~ 70% of all breast tumors [3–5]. Luminal A tumors are generally associated with a more favorable prognosis and typically express ER and PR. In contrast, luminal B tumors, while also expressing ER, display lower expression of PR and exhibit a higher rate of proliferation than luminal A tumors, resulting in a less favorable prognosis [3–5]. HER2 positive tumors, which comprise ~ 15% of all breast cancers, express high levels of HER2 but lack ER, and PR expression [3–5]. These tumors are associated with a less favorable outlook compared to luminal tumors. Triple-negative breast cancer (~ 15% of all breast cancers) lacks expression of ER, PR, and HER2, making targeted therapies ineffective and resulting in worse survival rates compared to other subtypes [3–5].

**Fig. 1:**
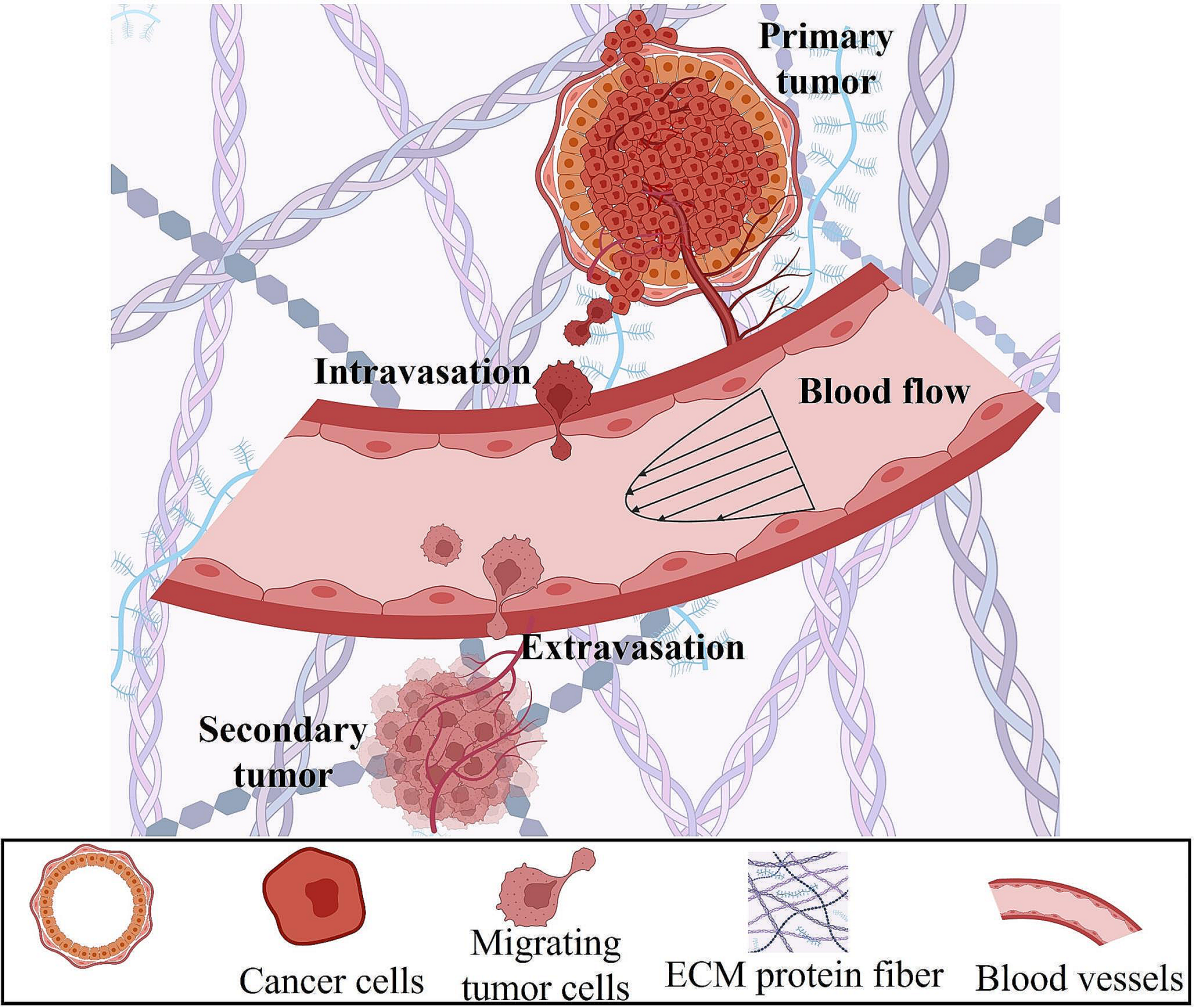
Schematic illustrating the multi-step process of breast cancer metastasis, with tumor cells escaping the primary tumor, entering the circulation, and extravasating to form a secondary tumor

**Fig. 2:**
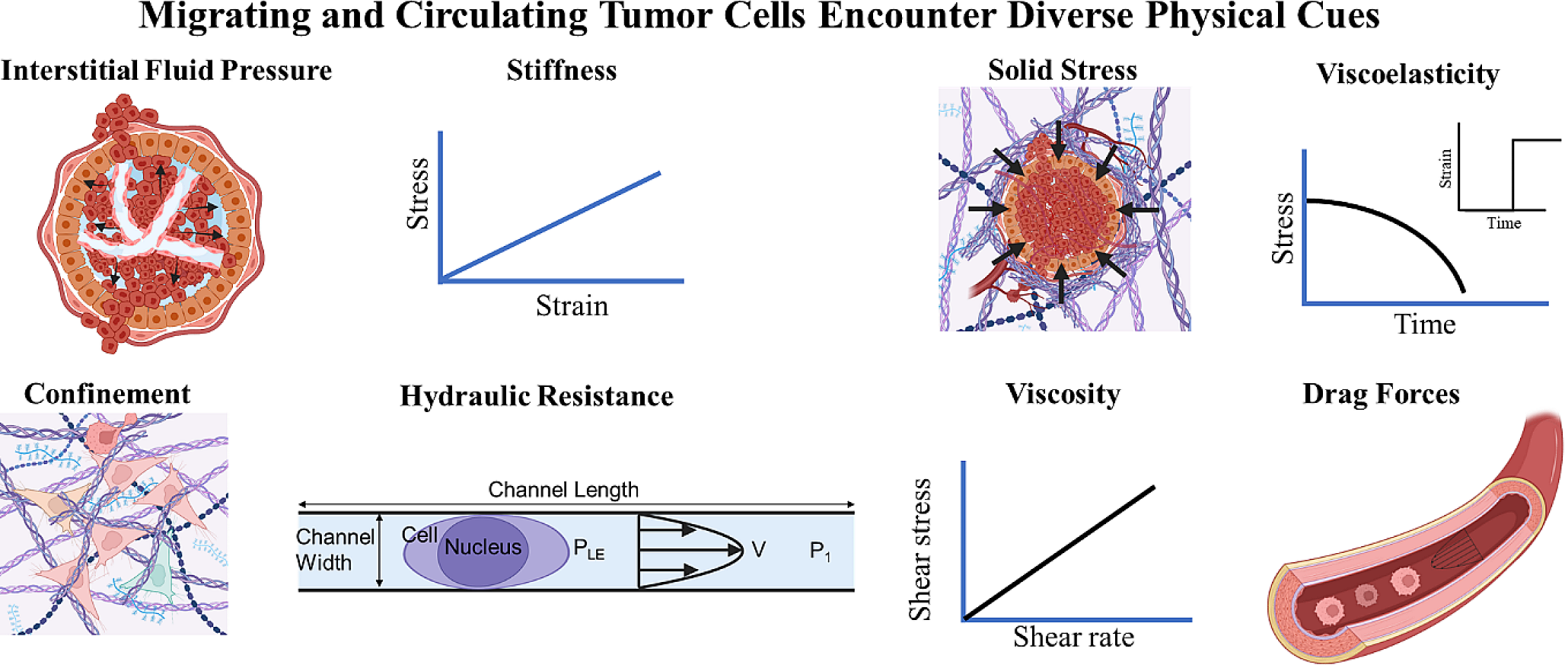
Throughout the metastatic cascade, breast tumor cells encounter a diverse array of physical cues that profoundly influence their behavior. These include interstitial fluid pressure, matrix stiffness, solid stress, viscoelasticity, confining 3D topographies, hydraulic resistance, extracellular fluid viscosity, and drag forces. P_LE_: hydraulic pressure at the cell leading-edge; V: fluid velocity; P_1_: upstream pressure

The lack of strategies to clinically interrupt and treat the metastatic spread of tumor cells accounts for the vast majority of breast cancer-associated deaths. This is supported by data showing that the 5-year survival rate for women diagnosed with distant-stage breast cancer is 29% versus 85–99% for those diagnosed with local- or regional-stage disease [6]. During metastasis, a tiny fraction of tumor cells splits away from the primary tumor region enters the circulation (intravasation) and exits (extravasation) to form a secondary tumor at a distant region (Fig. 1) [7, 8]. These migrating or circulating tumor cells are exposed to various physical cues, including interstitial fluid pressure, matrix stiffness, solid stress, viscoelasticity, confining 3D topographies, hydraulic resistance, extracellular fluid viscosity, and drag forces (Fig. 2; Table 1) [9, 10].

**Table 1:** Description of different physical cues experienced by breast cancer cells during tumor progression and metastasis

Physical stimuli		Definition
Interstitial fluid pressure (IFP)		The pressure exerted by the interstitial fluid on cancer cells
Extracellular matrix (ECM) stiffness		The resistance of the extracellular matrix to deformation when stress is applied
Solid stress		Compressive Stress: The force per area exerted by the surrounding tissue on tumors as they grow
		Residual Stress: The force per area generated due to intratumoral cell-cell and cell-ECM interactions
Viscoelasticity		A characteristic feature of living tissues that exhibit both viscous and elastic behavior
Cellular Confinement		Spatial restrictions imposed on cells during migration and metastasis
Hydraulic resistance		The external load that resists fluid movement
Viscosity		A measure of the fluid’s resistance to deformation under the influence of shear stress
Drag forces		Tangential (shear) or normal (pressure) forces exerted by the moving fluid on cells

Emerging evidence demonstrates that tumor cells possess the ability to sense and interpret these signals through mechanosensors and mechanotransducers, including mechanosensitive ion channels (e.g., ion channels that belong to the transient receptor potential (TRP) family), ion transporters, focal adhesions, cytoskeletal elements, nuclear proteins, and transcription factors. In response to physical stimuli, these molecules can initiate signal transduction, modulate the intracellular ion concentration (e.g., calcium, sodium, and chloride), influence cellular tension, volume, and shape, and trigger transcriptional changes, thereby contributing to therapeutic resistance and mediating profound changes in cellular functions, including division, and migration (see sections below). This review describes the contributions of the aforementioned physical cues to breast tumor progression and discusses the underlying mechanosensing mechanisms. In our opinion, unraveling the diverse mechanoresponses of breast tumor cells will facilitate the development of new therapeutic approaches to combat breast cancer progression.

## Interstitial fluid pressure

The hydrostatic and osmotic pressure differences between the vasculature and the surrounding tissue move fluid out from the blood vessels to the interstitium, allowing the supply of nutrients and oxygen to the resident tissue cells [11]. The lymphatic system serves the purpose of maintaining interstitial fluid homeostasis and removing unwanted waste products. As a result, the IFP of normal breast parenchyma is ~ 0 mm Hg [12]. The increased demands of growing tumors in nutrients and oxygen triggers the formation of new blood vessels, a process called angiogenesis. The tumors’ tortuous and hyperpermeable vessels [13–16] and the near absence of functional lymphatic vessels [17, 18] raise the interstitial fluid pressure (IFP) within breast tumors (Table 2). Using a small number of patients, Jain’s group has reported that IFP scales with breast tumor aggressiveness [19]. In this study, the IFP range was between 4 and 33 mm Hg, with the highest pressure measurements recorded in advanced breast tumors [19]. These findings have been corroborated by others, who have demonstrated elevated IFP in invasive ductal carcinomas relative to benign tumors or noninvasive carcinomas and a positive correlation between tumor size and IFP [12]. In addition to human breast tumors, IFP increases in experimental models of breast cancer, such as severe combined immunodeficient mice bearing highly invasive, triple-negative MDA-MB-231 human breast cancer tumors [20] and BN472 rat mammary carcinomas [21].

**Table 2:** IFP measurements in normal and abnormal breast tissues using the wick-in-needle technique

Breast Tissue	IFP (mmHg)			
	Human		Mouse	
Normal Breast Parenchyma	-0.3 ± 0.1 [12]		~ 0 [20]	
Benign conditions	0.4 ± 0.4 [12] 3.6 ± 0.8 [12]			
Breast Carcinoma	Non-invasive	-0.3 ± 0.2 [12]	hormone-independent LM3 murine breast cancer cells	2.9 ± 0.4 [54]
	Tumor stage (T2-T4)	15 ± 9 [19]	hormone-dependent MCF-7 human breast cancer cells	14 ± 10 [24]
	Invasive	29 ± 3 [12]	triple-negative MDA-MB-231 breast cancer cells	19.4 ± 3 [20]
			tripe-negativeMDA-MB-231 breast cancer cells	~ 11 [55]
			triple negative MDA-MB-468 breast cancer cells	~ 8 [55]

Micropuncture and wick-in-needle are the gold standard techniques for measuring IFP. Despite yielding the most reliable values and being less traumatic, micropuncture is limited by its depth of penetration which does not exceed 1 mm [11]. Thus, researchers gravitate towards the wick-in-needle technique which enables deeper penetration. Noninvasive methods for assessing IFP include intravoxel incoherent motion-diffusion weighted imaging [22], ultrasound poroelastography [23] and contrast-enhanced magnetic resonance imaging [24].

Although the IFP is relatively uniform throughout the breast tumor, it decreases significantly at its edge [25]. This pressure gradient triggers outward convection, creating a barrier to effective drug delivery [26, 27] and influencing breast cancer cell behavior (see also the “Drag forces” section below). A strategic way to circumvent the impaired transport of therapeutic agents is to reduce IFP and thereby improve tumor perfusion. This can be achieved using antiangiogenic agents which promote vascular normalization [28, 29]. For instance, the administration of the anti- VEGF-receptor-2 antibody DC101 enhances penetration of therapeutic molecules and nanomedicines up to the size of ~ 12 nm [30]. Abraxane, formulated with albumin-bound nanoparticles and the cancer chemotherapy agent paclitaxel, is a nanomedicine within this size range that has been effective in breast cancer treatments [30–32]. Targeting VEGF due to its over-secretion during tumor progression has also prompted the use of bevacizumab, an anti-human VEGF monoclonal antibody that reduces the growth of tumor vessels [33, 34]. Bevacizumab has been used in many breast cancer clinical trials in combination with chemotherapy. However, these combination therapies have not shown a significant increase in overall survival compared to chemotherapy alone [35–38]. This lack of significant improvement may be attributed to the activation of VEGF-independent angiogenic pathways, the recruitment of proangiogenic stromal cells, the induction of vasculogenesis or even the dose of the antiangiogenic agent used, which may reduce the size of blood vessel pores, further obstructing the delivery of this antibody [30, 39, 40]. In addition to antiangiogenic agents, certain chemotherapeutic agents have shown promising results in suppressing IFP. Taxanes, such as paclitaxel and docetaxel have been found to inhibit angiogenesis at low concentrations [41–44], and promote vascular decompression, effectively suppressing IFP in mouse mammary carcinoma [45]. These positive outcomes have been extended to breast cancer patients, where paclitaxel administration resulted in a ~ 40% reduction in IFP and improved tumor oxygenation [46]. However, it is important to note that not all chemotherapeutic agents have the same impact. For instance, doxorubicin does not elicit significant effects on oxygen levels and fails to ameliorate IFP in breast cancer patients [46].

Despite the well-established increase in IFP within breast tumors, our understanding of how breast cancer cells respond to this physical stimulus remains elusive due to the scarcity of reliable in vitro platforms that permit adjustment of pressure independent of changes in pH or the dissolved concentration of gases. Moreover, experiments are often performed under supraphysiological pressures [47–49] that are orders of magnitude larger than those sensed by tumor cells in vivo. Recent work has revealed that breast tumor cells possess the ability to detect subtle hydrostatic pressure changes, as low as 0.02 mmHg [50]. This extraordinary sensitivity is mediated by the activation of the mechanosensitive transient receptor potential cation channel subfamily M member 7 (TRPM7), which in turn triggers calcium ions (Ca^2+^) influx, thereby promoting a thicker actomyosin cortex [50]. Considering these intriguing findings, it is reasonable to expect that the substantially higher IFP levels found within tumors would have a profound impact on tumor cell behavior. Indeed, exposure of tumor cells to elevated, yet physiologically relevant, pressure, using a pressurized cultured system that maintains a constant pH, has been found to increase or decrease cell proliferation, depending on the cancer cell type [51]. Moreover, increased pressure has been shown to enhance cancer cell migration and resistance to the anticancer drug doxorubicin by upregulating the water channel aquaporin 1 [52] and the efflux transporter ABCC1 [53], respectively. Further research is needed to fully elucidate the effects of IFP on breast cancer metastasis as well as the mechanisms governing pressure-induced responses.

## Extracellular matrix stiffness

The extracellular matrix (ECM) is a key component of the tumor microenvironment, providing structural support and acting as a reservoir of cues, including biophysical signals such as matrix stiffness and viscoelasticity. The ECM of the healthy mammary gland includes glycosaminoglycans and proteoglycans (e.g., hyaluronan and versican), fibrillar collagens (e.g., type I and type III collagen), basement membrane proteins (e.g., collagen type IV and laminins), and fibronectin [56]. During breast cancer, tumor cells recruit resident fibroblasts [57], adipocytes [58], and/or bone marrow-derived mesenchymal stem cells [59], transforming them into cancer-associated fibroblasts (CAFs), which in turn reshape and remodel the tumor microenvironment. To induce this transformation, tumor cells secrete signaling molecules (e.g., Wnt7a [60], and osteopontin [61, 62]), and release exosomes containing miRNAs (e.g., miR-9 [63] or miR-125b [64]) and proteins like survivin [65].

Typically, CAFs overexpress alpha-smooth muscle actin (α-SMA), Vimentin, platelet-derived growth factor receptor α (PDGFRα), PDGFRβ, Tenascin-C, Caveolin 1, fibroblast activation protein (FAP), and ferroptosis suppressor protein 1 (FSP1) [66]. However, it is important to note that while these markers are commonly associated with CAFs, they are not limited exclusively to this cell type. Other cell types, such as normal fibroblasts, smooth muscle cells, and pericytes, also express them. In addition to their role in promoting breast cancer cell proliferation [67] and mediating inflammation [68], breast CAFs deposit collagen type I and fibronectin [58], secrete matrix metalloproteinases (MMPs) [69], and promote collagen crosslinking (e.g., via lysyl oxidase (LOX)) [70]. These alterations in the tumor ECM induce an increase in its stiffness, typically evaluated using techniques that either require direct contact with the sample (e.g., atomic force microscopy), or measure the tumor mechanical properties in a contact-free manner (e.g., shear wave elastography, magnetic resonance elastography) (Table 3). Besides CAFs, breast cancer cells can also trigger ECM stiffening by increasing the tension of ECM fibers [71]. The quantification of ECM stiffness can serve as a valuable diagnostic and prognostic marker for breast cancer [72–74], improving physicians’ clinical assessment of tumor status and their capability to predict patient outcomes.

**Table 3:** Comparative stiffness analysis between healthy and abnormal human breast tissues

Breast Tissue	Elastic modulus	Stiffness (kPa)	
Normal Breast Parenchyma	Young’s modulus	31.3 ± 1.6 ^d^ [104]	
		0.4 ^c^ [75]	
Benign conditions	Young’s modulus	34.8 ± 17.7^a^ [105]	
	Shear modulus	0.87 ± 0.15^b^ [106]	
		1.4 ± 0.5 ^b^ [107]	
Breast Carcinoma	Young’s Modulus	140.7 ± 58.5 ^a^ [105]	
		Non-invasive	42.8^a^ [108]
		Invasive ductal	174.4 ± 42^a^ [108]
			~ 3^c^ (invasive front) [75]
		Invasive lobular	208.2^a^ [108]
	Shear modulus	2.9 ± 0.3 ^b^ [106]	
		3.1 ± 0.7 ^b^ [107]	
Luminal breast cancer	Young’s Modulus	136.9 ± 57.2 ^a^ [109]	
		91.4 ± 30.7 ^a^ (Luminal A) [110]	
		108.2 ± 27.1 ^a^ (Luminal B) [110]	
HER2 positive breast cancer	Young’s Modulus	118.0 ± 32.1 ^a^ [110] 160.3 ± 56.2 ^a^ [109]	
Triple-negative breast cancer	Young’s Modulus	118.5 ± 30.8 ^a^ [110] 165.8 ± 48.5 ^a^ [109]	

^a^Shear wave elastography, ^b^ Magnetic resonance elastography, ^c^Atomic force microscopy, ^d^Virtual touch tissue imaging quantification

Stiffness measurements have demonstrated that the invasive front of HER2 positive and triple-negative human breast cancers is stiffer compared to luminal A and luminal B breast tumors [75]. Elevated stiffness promotes breast tumor progression by increasing angiogenesis and vascular permeability [76] as well as breast cancer cell proliferation [77], stemness [78], and metastasis [70]. Importantly, matrix stiffness can activate the epithelial to mesenchymal transition (EMT) program, resulting in a partial EMT state [79], which is distinguished by the simultaneous presence of both epithelial and mesenchymal traits, enabling a more efficient collective cell migration and invasion [80]. This stiffness-induced hybrid EMT state can be regulated at least in part by the transcription factor Twist1 [79]. In soft environments, Twist1 remains cytoplasmic by binding to Ras GTPase-activating protein-binding protein 2 (G3BP2) [79]. However, matrix stiffening promotes the dissociation of the Twist1-G3BP2 complex, and the translocation of Twist1 to the nucleus, where it initiates EMT, thereby promoting breast tumor invasion and metastasis [79]. In addition, breast cancer cells that express the EMT transcription factors Twist1, Snail1, and Six1 can enhance the migratory and metastatic potential of neighboring, non EMT tumor cells by activating the GLI-induced transcription factor in a non-cell autonomous manner [81].

Cells perceive elevated stiffness primarily through integrins, which promote cell spreading and Ras homolog family member A (RhoA)-dependent cytoskeleton tension [82–85, 77]. As a result, increased stiffness triggers the translocation of the transcription factor co-activators Yes-associated protein (YAP) and transcriptional coactivator with PDZ-binding motif (TAZ) to the nucleus where they interact with the transcriptional enhanced associate domain (TEAD) family of transcription factors to induce gene expression [85]. In contrast, low stiffness increases the activity of the Ras-related GTPase RAP2 which inactivates RhoA and induces the phosphorylation of the large tumor suppressor kinase 1/2 (LATS1/2). In turn, LATS1/2 phosphorylate YAP/TAZ to inactivate them, promoting their cytoplasmic localization [86].

Elevated YAP nuclear levels have been detected in CAFs within adenoma and carcinoma lesions in mice as well as in stromal fibroblasts of human breast cancer. Interventions aimed at reducing YAP levels or increasing their activity in CAFs provide evidence that YAP is essential for CAFs-dependent tumor cell invasion, matrix stiffening, and angiogenesis [87]. Interestingly, in contrast to CAFs, YAP nuclear intensity exhibits a significant decrease in both ductal carcinoma in situ and in invasive ductal carcinomas [88, 89]. This decrease is likely attributed to the loss of stress fibers and the reduction of nuclear area, which are observed in 3D environments but not on 2D surfaces [89]. The loss of YAP in breast cancer leads to increased tumor cell invasion, proliferation, resistance to chemotherapeutics, and protection against cell death, suggesting that YAP acts as a tumor suppressor [88].

Matrix stiffness has been shown to strongly influence the migration of breast cancer cells. Experiments conducted using a polyacrylamide-based microchannel device that allows independent control of substrate stiffness and channel dimensions revealed that optimal migration of triple-negative MDA-MB-231 and SUM159 human breast cancer cells occurs at intermediate stiffness [90]. This biphasic stiffness dependence finds its explanation in the motor-clutch model. According to this model, myosin motors, actin cytoskeleton, and cell adhesion molecules like integrins (clutches) cooperate and coordinate to facilitate cell migration [91–93]. The motor-clutch model predicts that cells generate maximal traction force at intermediate stiffness [92]. Moreover, it demonstrates that a change in the number of motors and clutches can alter the optimal stiffness for cell migration [92]. While these predictions have been experimentally confirmed [93], it has been observed that the optimal stiffness is often masked because, beyond a certain stiffness threshold, talin mediates adhesion reinforcement, leading to an increase in cell traction [94].

Substrate stiffness can also act as a guiding cue for migrating tumor cells. Research indicates that cells respond to stiffness gradients through a process called durotaxis (from Latin “durus” meaning hard and Greek “taxis” meaning arrangement), initially observed using fibroblasts [82]. While certain tumor cells, like MDA-MB-231 cells, display positive durotaxis by moving from soft (0.5 kPa), to stiff (22 kPa) substrates [95, 96], others, such as U-251MG glioblastoma brain tumor cells, undergo negative durotaxis, migrating from 22 kPa regions towards softer, 10 kPa, areas [96]. Recent work has significantly advanced our understanding of the intricate mechanisms that govern these diverse durotactic responses. By employing a combination of experimental and computational approaches, the Odde lab demonstrated that cells preferentially move toward areas of optimal stiffness, where they produce maximum traction forces [96]. For U-251MG cells, this optimal stiffness has been found to be ~ 10 kPa, while for MDA-MB-231 cells, it is ~ 20 kPa. Interestingly, negative durotaxis of U-251MG cells can be reversed by a partial reduction in myosin motors which increases the optimal stiffness of these cells. On the other hand, talin knockdown promotes negative durotaxis of MDA-MB-231 cells by suppressing adhesion reinforcement, which is observed on ~ 20 kPa substrates [96].

Therapeutic approaches aimed at targeting CAFs and their products show promise in suppressing breast tumor progression. Attractive targets include members of the LOX family, namely LOX and LOX-like 2 (LOXL2), which mediate collagen and/or elastin crosslinking [97, 98], thereby contributing to ECM remodeling in the tumor microenvironment. Chemical inhibition of LOX activity using β-Aminopropionitrile (BAPN) has been shown to increase tumor latency and decrease tumor incidence in the mouse mammary tumor virus (MMTV)-Neu model [70]. BAPN also re-sensitizes triple-negative breast cancer cells to chemotherapy as seen in different triple-negative breast cancer models, including chemoresistant xenografts, syngeneic tumors and PDX models [99]. Moreover, in a xenograft mammary 4T1 tumor model, it has been demonstrated that the concurrent administration of BAPN and the enzyme-responsive drug (NQO1-SN38), designed to target breast tumors with the topoisomerase I inhibitor SN38, results in a cooperative reduction in tumor growth [100]. The copper chelator tetrathiomolybdate, which blocks the copper-dependent catalytic activity of LOX, is presently undergoing a phase II study (NCT00195091) involving patients with breast cancer at moderate to high risk of recurrence. Furthermore, chemical or genetic inhibition of LOXL2 significantly reduces metastasis in orthotopic and transgenic breast cancer models [101]. Elevated LOXL2 expression in estrogen receptor-negative breast tumors is strongly associated with unfavorable outcomes, including poor prognosis, decreased overall survival, and reduced metastasis-free survival, suggesting that LOXL2 could serve as a valuable prognostic marker for metastasis in breast cancer cases. The antibody simtuzumab, targeting extracellular LOXL2, has undergone clinical trials in combination with gemcitabine for patients with metastatic pancreatic adenocarcinoma or with FOLFIRI (folinic acid, fluorouracil, and irinotecan) for patients with KRAS mutant colorectal cancer [102, 103]. However, the addition of these chemotherapeutics does not yield improved clinical outcomes, presumably because simtuzumab exclusively inhibits extracellular LOXL2. Phase I and phase II trials are also underway to assess the safety and pharmacokinetics of small molecule inhibitors of LOXL2, such as GB2064 (NCT04679870) and PXS-5382 A (NCT04183517). It is noteworthy that as of now, these inhibitors have not been tested for breast cancer treatment.

## Solid stress: compressive and residual stress

Breast cancer growth as well as collagen and hyaluronan deposition within the confined tissue environment results in the buildup of mechanical stress (aka solid stress) within tumors. This stress squeezes lymphatic and blood vessels, elevating IFP, hindering the effective delivery of chemotherapeutics, and promoting hypoxia [111, 112]. Two primary factors contribute to solid stress: compressive and residual stress [111, 113–115]. Compressive stress arises from the surrounding healthy tissue as it counteracts tumor growth. Residual stress is generated from intratumoral cell-cell, cell-ECM interactions, and consequently, persists even after tumor excision [115]. In human breast tumors, this stress is estimated to range from 10 to 19 kPa [115]. Although ultrasonography has enabled the in situ evaluation of 1D profile of solid stresses within glioblastoma tumors [111], the estimation of both the isotropic and anisotropic components of the stress tensor exerted on cells and tissues remained elusive until recently. This has been addressed through the use of multimodal intravital microscopy of deformable and adjustable in size fluorescently-labelled polyacrylamide beads that allow spatiotemporal measurements of solid stress applied to relatively small animal tumors in vivo [116]. This innovative technique has revealed that the solid stress imposed on murine breast tumors is ~ 2 kPa, a magnitude 5 to 8 times higher than the stress that individual cancer cells sense within the primary tumor [116]. Intriguingly, breast cancer cells in metastatic lung tumors experience higher magnitude of solid stress compared to those in the primary tumor, underscoring the key role of the local microenvironment in modulating solid stress during cancer progression [116]. Despite the significance of this method in measuring solid stress in vivo, its clinical applicability is hindered by the need for intravital window implantation.

In vitro and in vivo studies have provided valuable insights into how compressive stress affects breast cancer cell behavior. Initial studies using cancer cells embedded in non-degradable agarose gels showed that compressive stress exerted by the confining matrix on the mammary tumor spheroids suppresses proliferation and survival [117, 118]. However, more recently, it has been shown that tumor cells, including MDA-MB-231 and MCF7 breast cancer cells, display increased proliferation when subjected to confining stress from stiff (> 30 kPa), degradable, 3D, hyaluronan-based gels, compared to softer (~ 4 kPa) hydrogels [119]. These stiff gels activate the mechanosensitive ion channel (MIC) TRPV4, which upregulates the activity of the phatidylinositol 3-kinase(PI3k)/Akt pathway, triggering the expression of heat shock protein-(HSP-) 70, which protects against stress-induced cell death [119]. Moreover, HSP-70 enhances tumorigenicity and metastasis in murine models and promotes the expression of stemness markers (Nanog, Oct3/4, and SOX2) in stiff hydrogels by increasing the activity of the transcription factor signal transducer and activator of transcription 3 (STAT3) [119]. The disparity in findings across studies could stem from the characteristics of the hydrogel system used, such as its degradability and its initial stiffness. Additionally, differences in cell lines (e.g., P53 status) could lead to divergent cell responses to solid stress.

Furthermore, the application of external compressive stress of 0.77 kPa has yielded diverse migratory responses in breast cancer cells. While highly aggressive triple-negative 4T1 and MDA-MB-231 breast cancer cells show increased migration under this stress, non-metastatic, luminal A MCF7 breast cancer cells experience a significant reduction in motility [120]. Compression-induced invasion and migration of breast cancer cells are attributed to the activation of the MIC Piezo 1 [121] and the formation of leader cells, which possess filopodia at their leading-edge, thereby driving persistent and directional motility [120]. Interestingly, the application of low-magnitude compressive stress (0.03 kPa) on weakly adhesive MDA-MB-231 breast cancer cells decreases their motility, highlighting the critical role of cell-matrix interactions in stress-dependent cell responses [122].

Besides its role in regulating cell division, viability, and motility, compressive forces can also influence gene expression [123]. Compression- or actomyosin-mediated nuclear flattening alters the permeability across nuclear pore complexes, resulting in active nuclear import of key transcription regulators such as YAP, SMAD3, Twist1, and Snail [124, 125]. Compressive stress also induces promoter hypermethylation of microRNA-9 (miR-9) precursors, leading to miR-9 downregulation, which increases VEGFA expression [126]. This mechanism, which is observed in breast CAFs, as well as in luminal B (BT-474) and triple-negative (MDA-MB-231) breast cancer cells, but not in HER2 (SK-BR-3) and luminal A (MCF7) tumor cells, may contribute to angiogenesis in breast tumors [126].

Interventions aimed at blocking collagen and hyaluronan production, such as a neutralizing antibody against TGF-β [127], the angiotensin inhibitor losartan [128–131], or the antifibrotic drugs tranilast and pirfenidone [132–134] have shown promising results in alleviating solid stress and increasing breast tumor blood supply and drug delivery. As a result, these interventions boost the efficacy of chemotherapeutic agents, markedly suppressing breast tumor growth in preclinical animal models of breast cancer [127–132, 134]. Importantly, a clinical study has demonstrated that combining radiotherapy with higher doses of the TGFβ-blocking antibody fresolimumab (10 mg/kg) increases overall survival significantly in metastatic breast cancer patients compared to those undergoing radiotherapy and receiving lower doses of fresolimumab (1 mg/kg) [135]. Furthermore, in a phase II study, the combination of chemotherapy (albumin-bound paclitaxel and gemcitabine) along with pegvorhyaluronidase alfa (PEGPH20), an agent designed to degrade hyaluronan, has been shown to improve progression-free survival in pancreatic cancer patients, especially those with hyaluronan-high tumors [136]. However, the potential efficacy of such approach for breast cancer patients remains to be determined.

## Viscoelasticity

Various living tissues and natural ECMs exhibit both viscous (fluid-like) and elastic (spring-like) behavior. While purely elastic materials recover their shape following stress application, viscoelastic materials display (a) a time-dependent stress decrease in response to a constant strain (aka stress relaxation), (b) a time-dependent strain increase under constant stress (aka creep), and (c) an energy dissipation and a delayed response as the materials undergo loading and unloading (aka hysteresis) [137]. Stress relaxation tests have revealed that the time it takes for biological tissues to relax to half the magnitude of the initially applied stress varies significantly. For instance, the relaxation time can range from seconds in the brain, to minutes in the liver, and hours in the skin, with faster stress relaxation indicating increased viscoelasticity [138]. Although the investigation of the role of viscoelasticity in health and disease has recently just begun, techniques such as shear wave elastography, and magnetic resonance elastography have demonstrated that malignant breast tumors exhibit more viscoelastic behavior relative to benign and normal tissues [139–141]. Moreover, the indentation method has revealed that human breast tumors exhibit stress relaxation half time of ~ 10 s [142].

The Chaudhuri lab investigated the effects of viscoelasticity on tumor cell behavior using alginate gels with varying stress relaxation times. They found that the 2D motility of HT-1080 fibrosarcoma cells, MDA-MB-231 breast cancer cells, and normal breast epithelial MCF-10 A cells is faster on fast- (~ 100 s) relative to slow-relaxing (~ 2,000 s), soft (2 kPa) substrates [143]. This effect is attributed to the increased formation of nascent adhesions and filopodia at the cell periphery and cell front, respectively [143]. In addition to promoting cell migration, fast- (~ 60 s) but not slow- (~ 6,000 s) relaxing hydrogels allow breast cancer cells to grow in size and divide in 3D confining environments. Cell growth, controlled by sodium-hydrogen ion exchangers (NHEs), induces MIC-dependent activation of the PI3K/Akt pathway, which in turn supports the cytoplasmic localization of the cell cycle inhibitor p27^Kip1^, resulting in tumor cell proliferation [142]. Furthermore, measurements have shown that human breast tumors display plastic deformation (i.e., permanent deformation when subjected to external forces) [144], likely due to the increased collagen concentration and crosslinking observed in the breast tumor microenvironment which can enhance plasticity [145]. To model this plastic behavior, 3D hydrogels composed of reconstituted basement membrane and alginate are used. Increased plasticity has been shown to facilitate protease-independent migration of breast cancer cells by promoting the formation of invadopodia protrusions, which in turn generate contractile forces to create migratory paths for the cells [144].

## Cellular confinement

Microscopy techniques, including intravital microscopy, have revealed that metastatic cancer cells traverse microenvironments that impose different levels of confinement on cells. Examples of such environments are microvessels with diameters smaller than the size of tumor cells [146], narrow (~ 1–5 μm-sized) gaps between endothelial cells [147], micropores with diameters ranging from 1 to 20 μm [148], ECM fibers [149], and longitudinal, channel-like tracks with widths ranging from 3 to 30 μm [150]. Advances in patterning, materials and microfabrication techniques have enabled researchers to isolate and investigate the effects of confinement on tumor cell behavior. In vitro models to study confinement-induced responses include micropatterned lines [151], 2D micropatterned substrates [85], uni-axial compression [152], polydimethylsiloxane [153–156]- or polyacrylamide [90]-based microchannel devices, microniches [157, 158], and natural hydrogels [148, 159]. These versatile tools enable in-depth studies of cellular responses to 1D, 2D or 3D confinement [85, 148, 152, 159, 160], moderate or tight confinement [157, 158, 153–155, 160], short- or long-term confinement [153–155, 160], and confining pores or channels [148, 159, 153–155]. Additionally, recent research has delved into the interplay between confinement and other physical cues (e.g., stiffness [90], viscoelasticity [142], and viscosity [161]) in relation to cancer cells, revealing their combined contributions to different cell processes.

It is well established that breast cancer cells alter their migration modes and mechanisms to adapt to 3D confinement. Inhibition of key regulators of 2D cell locomotion such as actin polymerization, cell-matrix adhesion, and myosin contractility fail to block confined migration of MDA-MB-231 breast cancer cells [153], suggesting the existence of alternative mechanisms facilitating their movement. The Osmotic Engine Model (OEM) predicts that confined cell migration is facilitated by directed water permeation driven by a gradient of aquaporins, ion transporters, and ion channels [162]. Consistent with the OEM, experiments have demonstrated that in confinement, aquaporin 5 and the sodium-hydrogen exchanger 1 (NHE1) localize at the cell front, promoting isosmotic cell swelling [162]. On the other hand, aquaporin 4 and the SWELL1 chloride channel (LRRC8A) accumulate at the cell rear mediating cell shrinkage [163]. Dual depletion of NHE1 and SWELL1 markedly suppresses breast cancer cell migration, extravasation, and metastasis, underscoring the pivotal role of this migration mechanism in breast cancer progression [163]. Furthermore, while in 2D/unconfined environments breast cancer cells maintain a mesenchymal migration phenotype characterized by actin-rich protrusions and adhesion to the substrate [7], in confinement, cells switch to an amoeboid/bleb-based migration mode [152, 164, 165]. Amoeboid cells exhibit weak adhesions, show high dependence on actomyosin contractility, and utilize spherical bulges known as membrane blebs for efficient migration, invasion, and extravasation [166–168]. Amoeboid-based migration of breast cancer cells has been found to correlate with lymph node metastasis [169].

Tumor cell migration speed scales with pore size in 3D hydrogels. However, in the absence of matrix degradation, pore sizes smaller than 7 µm^2^ impede cellular locomotion because the nucleus, which is the stiffest and largest cell organelle, acts as a rate-limiting barrier to migration [170–172]. Consistent with these findings, migration studies conducted in confining microenvironments have demonstrated an inverse correlation between nuclear rigidity or nuclear volume expansion with migration efficiency [173–177]. The nuclear lamina, which lines the inner surface of the nuclear envelope, is the main determinant of nuclear stiffness, providing structural support to the nucleus. It is composed of A- (A, C, C2) and B- (B1, B2, B3) type lamins, a mesh-like network of intermediate filament proteins [175, 178, 179]. Lower levels of lamin-A induced by the PI3K/Akt pathway facilitate nuclear deformation in confinement, support breast cancer cell invasion and associate with reduced disease-free survival [174]. B-type lamins can also enhance nuclear stiffness, thereby impacting confined migration [180, 181]. Other factors contributing to nuclear rigidity include the chromatin organization [182] and the LINC (Linker of Nucleoskeleton and Cytoskeleton) complex [183]. It is worth noting, that in addition to nuclear stiffness, A-and B-type lamins regulate other hallmarks of cancer, including cell proliferation and chromatin organization [184–186], underscoring their multifaceted role in tumor progression. Importantly, high lamin-B1 has been found to predict unfavorable outcomes in patients with different types of cancer [187].

Confinement exerts stress on the nucleus, triggering the formation of nuclear protrusions (aka nuclear blebs), which lack lamin B1 and nuclear pores and lead to transient ruptures of the nuclear envelope [188–190]. Actomyosin contractility can compromise nuclear integrity by squeezing the nucleus dorsoventrally [191, 192], pulling it [193] or promoting the nuclear influx of cytoplasmic constituents, which in turn pressurizes the nucleus, leading to its rupture [155]. Seminal studies have demonstrated that nuclear ruptures occur at sites characterized by pronounced curvature [194], leading to DNA damage and genomic instability [188–190]. These adverse effects stem from the entry of the cytoplasmic exonuclease TREX1 into the nucleus, which induces DNA cleavage [195], as well as the translocation of DNA repair factors such as Ku70, Ku80, and BRCA1 to the cytoplasm [190, 194]. Nuclear deformation can promote DNA damage even without instances of nuclear envelope ruptures. This type of DNA damage is more evident in MDA-MB-231 or BT-549 breast cancer cells, manifesting at replication forks and causing replication stress [196]. Although confinement suppresses MDA-MB-231 cell proliferation [160], it proves ineffective in inducing death in these cells as they harbor mutations in the gene encoding P53 [160]. P53 mutations are highly prevalent in breast tumors [197] potentially granting a survival advantage to breast cancer cells as they navigate mechanically challenging microenvironments.

Accumulating evidence suggests that tumor cell exposure to confinement can promote breast cancer progression. The invasive front of confined breast tumors exhibits pronounced nuclear deformation, nuclear rupture, and DNA damage [195]. A key player in the formation of invasive breast tumors is TREX1, which mediates confinement-induced DNA damage, collagen degradation, and the acquisition of a hybrid epithelial-mesenchymal phenotype [195]. Additionally, confinement triggers resistance to chemotherapeutics [198] and to programmed cell death triggered by loss of adhesion (aka anoikis) [199] which in turn facilitates breast cancer metastasis [199]. Collectively, confinement is a pathophysiologically relevant cue that can influence tumor progression significantly by altering the modes and mechanisms of cancer cell migration, promoting genomic instability, and inducing gene expression changes, which trigger EMT and provide resistance to therapeutic agents.

## Hydraulic resistance

Tumor cells migrate through confining, water impermeable channels by generating pressure at their leading-edge (P_LE_), which enables them to push the column of fluid ahead of them (Fig. 2). Assuming that cells are impermeable to water, P_LE_=P_1_ + < v > AR, where P_1_ is the upstream pressure (Fig. 2), <v > is the average fluid/cell velocity (since the fluid moves at the same speed as the cell), A is the cross-sectional area and R is the hydraulic resistance (i.e., the external load that resists cell/fluid movement) which, for rectangular channels, is proportional to the channel length *L.* This equation implies that if < v>, P_1_ and A are constant, it becomes easier for cells to migrate through channels with lower resistance, as they would need to generate less P_LE_. Indeed, when neutrophil-like cells (HL-60 cells) encounter channels with varying levels of hydraulic resistance, they preferentially select the path of least resistance. Intriguingly, when the hydraulic resistance is infinite, nearly all cells move towards the lower hydraulic resistance geometries [200].

MDA-MB-231 breast cancer cells, much like immune cells, demonstrate a preference for low resistance channels [50]. Using trifurcating Ψ-like microchannels, the Konstantopoulos lab studied how tumor cells respond to channels with different resistances. Their research revealed that the branch channel with the higher resistance triggers the TRPM7-mediated influx of Ca^2+^, resulting in the local formation of a thicker cortical actin network enriched with elevated levels of myosin II-A. This increase in actomyosin contractility guides cells towards low resistance channels [50]. Tumor cell migration along the path of least resistance is energetically favorable [201]. However, increasing cell compliance or reducing matrix stiffness suppresses energetic costs, blinding MDA-MB-231 cells to hydraulic resistance and enabling them to navigate through high-resistance migratory paths that would otherwise demand more energy [201]. Hydraulic resistance also alters the migration phenotype of confined MDA-MB-231 cells [202], by inducing the transition from an amoeboid phenotype to a mesenchymal phenotype [202]. This switch is controlled by TRPM7-dependent Ca^2+^ influx and requires actin polymerization, myosin recruitment and the formation of focal adhesions at the interface between the cell and the lateral channel walls. This redistribution of actomyosin contractility decreases cortical contractility, promoting amoeboid to mesenchymal transition [202]. Although the magnitude of hydraulic resistance experienced by migrating breast cancer cells in vivo is currently unknown, mathematical modeling indicates that depending on the interstitial fluid viscosity and matrix permeability, the hydraulic resistance within 3D matrices could potentially match or even exceed that observed in microchannels [50, 203]. This suggests that hydraulic resistance could significantly impact cell migration and decision-making in vivo.

## Extracellular fluid viscosity

In basic cell culture, the medium typically used matches the viscosity of water (~ 0.7 cP at 37 ^o^C). However, in vivo, the viscosity of extracellular fluids is higher than 0.7 cP (Table 4). Extracellular fluid viscosity is further elevated in tumors as recently demonstrated in a study that employed new viscosity-sensitive fluorescent probes for noninvasive imaging of murine breast tumors [204]. Shear wave elastography also showed that malignant breast tumors display a shear viscosity ~ 3 times higher than benign tumors and ~ 6 times higher than normal breast tissue (8.22 Pa∙s, 2.83 Pa∙s, 1.41 Pa∙s, respectively) [140]. This increase in extracellular viscosity can be attributed to the accumulation of ECM degradation products [205]or macromolecules (e.g., Mucins [206]) caused by the collapse of the lymphatic drainage system [17, 18]. Furthermore, clinical studies have shown that increased plasma viscosity correlates with poor survival in breast cancer patients [207]. Despite these findings, the impact of fluid viscosity on breast cancer progression remains largely unexplored. Recent work has shown that elevated viscosity increases the migration of both cancerous and non-cancerous cells [161, 208, 209]. This finding is counterintuitive, as higher viscosity is known to suppress the movement of particles in fluids.

**Table 4:** Viscosity measurements of different extracellular fluids

Extracellular Fluid	Range of viscosity (cP)	Range of shear rates (s^− 1^)	Fluid behavior
Synovial fluid	~ 70–900 [210]	10–250 [210]	Shear thinning
Cerebrospinal fluid	~ 0.7-1 [211]	25 − 1,460 [211]	~Newtonian
Gastric mucus	~ 500-7,000 [212]	1.15-46 [212]	Shear thinning
Blood	~ 3–5 [213]	1-100 [213]	Shear thinning

Cells sense elevated viscosity through their actin cytoskeleton, which rapidly reorganizes into a dense network that polarizes and activates NHE1 [161]. In turn, NHE1 promotes cell volume expansion, which increases membrane tension, resulting in TRPV4-mediated calcium influx and the activation of actomyosin contractility [161]. Moreover, elevated viscosity suppresses membrane ruffling, allowing the cell membrane to stay adjacent to the substrate, thus increasing integrin-substrate engagement [208]. These viscosity sensing mechanisms enhance cell traction force, promote cell spreading, and allow breast cancer cells to migrate faster on 2D substrates and in confining microchannels [161, 208]. Furthermore, breast cancer cells develop YAP-dependent viscous memory that enhances their migration in zebrafish, extravasation in chick embryos and lung metastasis in mice [161]. These studies suggest that the elevated extracellular fluid viscosity within breast tumors triggers cancer cell invasion and metastasis. However, it remains unclear how this physical signal affects other hallmarks of cancer, including drug resistance and angiogenesis.

## Drag forces: shear and pressure drag

Pressure gradients are highly prevalent within the human body, serving as the driving force behind fluid flow, including transmural, interstitial and blood flow. Moving bodily fluids display significant disparities in velocities, often differing by several orders of magnitude. Techniques such as fluorescence recovery after photobleaching and dynamic contrast-enhanced magnetic resonance imaging (MRI) have demonstrated that interstitial fluid generally moves at a relatively slow velocity, ranging from 0.1 to 10 μm/s, depending on the species under consideration [214]. In the context of cancer, the pressure buildup within the tumor relative to the surrounding tissue leads to a notable increase in interstitial flow, typically by 3–5 times [215]. Blood flow is much faster, reaching speeds of several mm/s in capillaries or cm/s in veins and arteries [216–218].

Moving fluid is known to exert drag forces on tumor cells in the direction of flow due to the combined effects of shear and pressure forces. While shear forces act tangentially to the cell’s surface, pressure drag acts normal to it. The shear stress, representing shear force per unit area, is particularly elevated near the vessel wall with values ranging from 1 to 4 dyn/cm^2^ in veins, and 4–30 dyn/cm^2^ in arteries [219]. As cells attempt to invade a blood vessel, they encounter shear forces acting on them. The diminished shear sensitivity of tumor cells like MDA-MB-231 cells or HT-1080 fibrosarcoma cells allows them to enter high-shear environments [220]. However, ectopic expression of TRPM7 restores their shear sensitivity, leading to a marked reduction in intravasation and metastatic lesion formation [220]. TRPM7 elicits its effects by triggering Ca^2+^ influx in response to fluid shear, thereby activating RhoA-dependent contractility and the calmodulin/IQGAP1/Cdc42 pathway [220]. This shear-sensing mechanism allows cells to reverse their migration direction, effectively avoiding entry into high-shear environments [220].

The increased shear stress in the circulatory system can pose a threat to circulating tumor cells (CTCs), triggering apoptosis or necrosis [221, 222]. Although breast cancer cell lines exhibit increased resilience to physiologically relevant shear stress compared to normal epithelial cells [223], lamin A/C knockdown exacerbates shear stress-mediated tumor cell death [223], suggesting that elevated lamin A/C levels could aid breast CTCs in withstanding shear forces in the circulation. CTCs that survive the harsh conditions induced by elevated shear stress acquire stem cell-like properties and display a mesenchymal phenotype [224, 225]. These shear stress-induced changes involve the activation of Jun N-terminal kinase (JNK) signaling and the downregulation of extracellular signal-related kinase (ERK) and glycogen synthase kinase (GSK)3β [224, 225].

The impact of shear stress on cell migration varies depending on the specific breast cancer cell line. Highly invasive triple-negative MDA-MB-231 cells respond to 15 dyn/cm² by increasing their migration velocity, whereas normal MCF-10 A epithelial cells and less aggressive triple-negative MDA-MB-468 breast cancer cells show only slight or no increase in motility under the same conditions, respectively [226]. MDA-MB-231 cells even respond to lower shear stress values (1.8 dyn/cm^2^) by adhering more to the substrate and reorienting the Golgi marker GM130 in the direction of flow [227]. Shear stress regulates breast cancer cell adhesion to endothelial cells. Optimal tumor cell adhesion is observed under low flow conditions, typically encountered in the venous but not arterial system [228, 229]. However, efficient extravasation also requires remodeling of the endothelium, a process that occurs at higher blood flow velocities and involves the engulfment of tumor cells by endothelial cells [229]. Thus, an optimal flow velocity range of 400–600 μm/s has been found to favor both tumor cell arrest within vascular regions and endothelial remodelling, leading to extravasation and metastasis [229].

While the effects of shear forces on tumor cell behavior are well-established, the impact of pressure drag on these cells remains largely unexplored. Computational fluid dynamics simulations have revealed that the pore size within a 3D matrix governs the relative magnitude of shear and pressure drag acting on a cell [230]. These simulations indicate that within highly confined matrices, such as those encountered in vivo, the force resulting from the pressure drop across the cell dominates over the total shear force [230]. Hence, in these microenvironments, pressure drag accounts for the majority of the total drag experienced by the cell. By employing 3D hydrogels typically characterized by small, micron-sized pores, the Kamm and Swartz labs have shown that (patho)physiologically relevant flow velocities can trigger migration of MDA-MB-231 in the upstream (against the flow) and downstream (with the flow) direction [231, 232]. Downstream migration is mediated by self-secreted ligands, specifically CCL19 and CCL21, which bind to the chemokine receptor CCR7 [233]. These ligands are distributed downstream due to convective flow, resulting in an autologous chemotaxis mechanism, which can direct tumor cells towards the draining lymphatics, facilitating their metastatic spread [233]. In contrast, upstream migration of tumor cells is regulated by focal adhesions, which polarize at the upstream side of the cell [234]. Cell migration against the flow can be observed in high-density cultures [234]. This behavior is consistent with results obtained from densely seeded suspensions of MDA-MB-231 cells in collagen gels, where cells tend to stay in high-pressure environments and do not move with the flow [235, 236]. These intriguing findings imply that the elevated IFP within the tumor may act as an anti-metastatic signal, hindering tumor cell escape. Furthermore, flow conditions that promote invasion of MDA-MB-231 aggregates in collagen gels trigger the upregulation of mesenchymal (Vimentin and Snail1) and epithelial (E-Cadherin and keratin-8) genes [237]. Depletion of Vimentin nearly abolishes flow-induced invasion [237].MDA-MB-231 cells also undergo phenotypic transitions in 3D hydrogels under the influence of physiologically relevant flows. While in the absence of flow, cells migrate using a mesenchymal phenotype, cell exposure to flow promotes a faster amoeboid-based migration [238]. Collectively, these findings highlight the significant influence of pressure-driven flow fields on various aspects of breast cancer progression.

## Conclusions and future directions

While the existence of physical stimuli within the breast tumor microenvironment and throughout the metastatic cascade has been acknowledged for several decades, our understanding of their role in breast cancer remains incomplete. Recent advances in bioengineering have provided powerful tools to tune biophysical and topographical cues of the microenvironment, opening up new avenues to explore how these signals contribute to tumor progression. However, these techniques often permit the investigation of only one factor at a time, hindering our ability to unveil the intricate interplay of various physical factors in cancer. Furthermore, little is known about the role of physical signals in the progression of different breast cancer molecular subtypes. Tailoring treatments according to the distinct features of the breast tumor microenvironment and its interactions with different molecular subtypes of breast cancer may facilitate the development of personalized medicine approaches.

Although a plethora of tumor cell mechanosensors have been identified, including focal adhesions, ion channels, actin cytoskeleton, nuclear proteins, and transcription factors, more research is needed to uncover how breast cancer cells convert biophysical signals into biochemical cues to mediate short- and long-term mechanoresponses **(**Table 5**)**. Gaining a deeper understanding of the crosstalk between different mechanosensors may inspire the development of new therapeutic strategies to block tumor progression. To date, the focus of new drug development and therapies has primarily revolved around targeting tumor growth, often overlooking metastatic spread, which accounts for the vast majority of breast cancer-associated deaths. Given the established role of confined cell migration in breast cancer metastasis [239], it is critical to identify interventions that can target and modulate cell motility.

**Table 5:** Influence of physical cues on tumor progression

Physical cue	Mechanosensing mechanisms and their impact on tumor progression
IFP	- Activates TRPM7, leading to calcium influx and thicker actomyosin cortex [50] - Upregulates aquaporin 1 to enhance tumor cell migration [52] - Regulates cancer cell proliferation [51] - Triggers ABCC1-dependent drug resistance [53]
ECM stiffness	- Promotes MMP-mediated angiogenesis and increases vascular permeability [76] - Triggers focal adhesion formation, increases Rho activity and promotes tumor progression [77] - Upregulates integrin-linked kinase (ILK) to induce cancer stem cell development [78] - Regulates the TWIST1-G3BP2 pathway to initiate EMT, invasion and metastasis [79] - Controls tumor cell migration efficiency and direction [90, 96] - Induces YAP translocation to the nucleus which mediates the pro-tumorigenic functions of CAFs [85, 87]
Solid stress	- Regulates tumor growth and survival [117–119] - Activates Piezo 1 and the TRPV4-PI3K/Akt pathway [119, 121] - Upregulates HSP-70 to promote stemness, cancer cell survival and metastasis [119] - Regulates cancer cell migration [120] - Induces VEGFA expression [126] - Regulates active and passive nucleocytoplasmic transport [124, 125]
Viscoelasticity	- Increases tumor cell migration on soft substrates [143] - Promotes the formation of nascent adhesions and filopodia protrusions on soft substrates [143] - Enables tumor cell proliferation in 3D environments [142] - Activates MICs which control the PI3K/Akt-p27 pathway [142]
Cellular confinement	- Polarizes aquaporins, ion channels and ion transporters which facilitate cancer cell migration, extravasation and metastasis [162, 163] - Triggers nuclear envelop rupture, nuclear-cytoplasmic exchange of material and DNA damage [188–190] - Activates P53-dependent DNA damage responses [160] - Suppresses YAP activity [160] - Reduces proliferation [160] - Promotes the transition from a mesenchymal to an amoeboid phenotype [152, 164, 165] - Triggers resistance to chemotherapeutics [198] - Induces resistance to anoikis [199] - Promotes cancer cell invasion and metastasis [195, 199]
Hydraulic resistance	- Activates TRPM7 [50] - Influences tumor cell decision making in confinement [50, 201] - Promotes the transition from an amoeboid to a mesenchymal phenotype [202]
Viscosity	- Induces actin remodelling which activates the NHE1-TRPV4-RHOA-Myosin II pathway [161] - Suppresses membrane ruffling [208] - Promotes cancer cell invasion, migration, extravasation and metastasis [161, 208, 209]
Drag forces	- Activate TRPM7-RhoA-Myosin II and calmodulin-IQGAP1-Cdc42 pathways [220] - Control cancer cell intravasation [220] - Activate JNK signaling and suppress ERK-GSK3β [224, 225] - Compromise cell survival [221, 222] - Regulate tumor cell adhesion to the endothelium and extravasation [228, 229] - Control migration direction [231, 232] - Upregulate mesenchymal and epithelial genes [237] - Promote the amoeboid mode of migration [238]

Physical signals hold significant potential as valuable diagnostic and prognostic markers for breast cancer. For example, magnetic resonance elastography has proven effective in identifying malignant tumors by detecting alterations in stiffness within the breast tissue [74, 240]. Integrating techniques used to assess the biophysical characteristics of the tumor microenvironment with image-based tools (mammography, MRI and ultrasound), and machine learning algorithms could further accelerate the development of advanced prognostic and diagnostic tools for breast cancer. Furthermore, recent advancements in microfluidics have led to the development of an assay that screens potential antimetastatic drugs and predicts the metastatic propensity of isolated breast cancer cells [239]. This technique has the potential to complement existing diagnostic assays and determine whether a patient is at an increased risk of metastasis [239]. In conclusion, a comprehensive understanding of the implications of the physical traits of breast cancer holds significant promise for driving advancements in diagnosis, prognosis, treatment selection, and ultimately improving patient outcomes.

## Data Availability

Not applicable.
